# Supplementary material for: Secretion-based production of prolyl-hydroxylated human type III collagen in scalable Physcomitrella photobioreactors
Source: Plant Cell Rep. 2026 Jan 28;45(2):45. doi: 10.1007/s00299-026-03727-7 (PMC12852254; doi:10.1007/s00299-026-03727-7)
Supplement: Supplementary file 1 — Supplementary file1 (PDF 1656 KB) [file 299_2026_3727_MOESM1_ESM.pdf]

Supplementary Information

Secretion-based production of prolyl-hydroxylated human type III collagen in scalable Physcomitrella photobioreactors

Lennard L. Bohlender[1], Juliana Parsons[1], Antonia Mitgau[1], Sebastian N. W. Hoernstein[2], Giovanna Grigolon[3], Bernhard Henes[3], Eva L. Decker[1], Ralf Reski[1,4,5,*]

[1]Plant Biotechnology, Faculty of Biology, University of Freiburg, Schaenzlestr. 1, 79104 Freiburg, Germany

[2]Biochemistry and Functional Proteomics, Faculty of Biology, University of Freiburg, Schaenzlestr. 1, 79104 Freiburg, Germany

[3]Mibelle Group Biochemistry, Mibelle AG, Bolimattstr. 1, 5033 Buchs, Switzerland

[4]CIBSS – Centre for Integrative Biological Signalling Studies, University of Freiburg, Schaenzlestr. 18, 79104 Freiburg, Germany

[5]Cluster of Excellence *liv*MatS at FIT – Freiburg Center for Interactive Materials and Bioinspired Technologies, University of Freiburg, Georges-Köhler-Allee 105, 79110 Freiburg, Germany

***Corresponding author**: ralf.reski@biologie.uni-freiburg.de

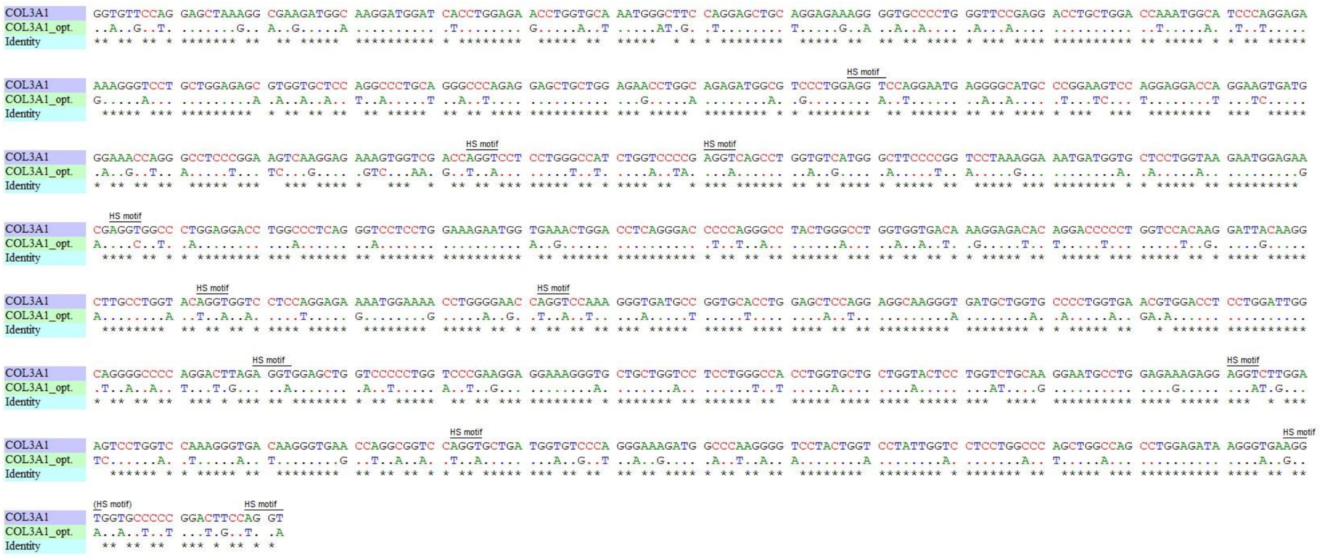

Supplementary Figure S1 Alignment of the selected coding region (nucleotides 1266–2367) of the human COL3A1 CDS (COL3A1, NM_000090.4) and the codon- and splice site-optimized COL3A1 sequence (COL3A1_opt.) used for generation of collagenproducing Physcomitrella lines

The CDS of the human collagen α1(III) chain (COL3A1) was obtained from NCBI (NM_000090.4). The region coding for the selected recombinant collagen polypeptide is depicted, aligned with the corresponding sequence after *in silico* codon and potential heterosplice sites optimization. Codon optimization according to Physcomitrella codon usage was carried out by Thermo Fisher Scientific. In addition, underrepresented codons (as listed in **Supplementary Table S1**) were substituted with corresponding, overrepresented alternatives using the physCO online tool (available at www.plant-biotech.uni-freiburg.de). To prevent potential heterosplicing, nucleotides within the heterosplicing recognition motif AGGT (HS motif, Top et al. 2021) were silently mutated *in silico*. The resulting optimized sequence (COL3A1_opt.) encodes the original amino acid sequence and was modified exclusively at the nucleotide level.

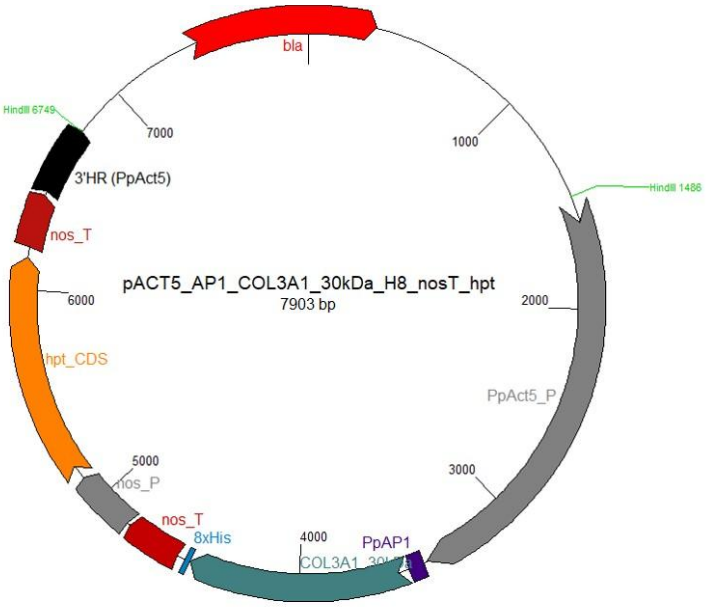

Supplementary Figure S2 Schematic representation of the pJET1.2-based vector containing the expression construct used to generate recombinant collagenpolypeptide producing Physcomitrella lines

The codon- and splice-site optimized collagen polypeptide CDS including the CDS for the aspartic protease signal peptide PpAP1 (Pp6c5_10120V6.1; Schaaf et al. 2004) and an 8xHistag were cloned into an pJET1.2-based expression vector containing the Physcomitrella Actin5 (PpActin5) promoter (Weise et al. 2006; Niederau et al. 2024) and the nos terminator (nos_T). For selection purposes this vector additionally contained a hygromycin selection cassette coding for a hygromycin B phosphotransferase (hpt; Decker et al. 2015) under the control of a nos promoter (nos_P) and a nos terminator. Targeted genome integration via homologous recombination was facilitated by a 327 bp long 3´ homologous region of the 3´ UTR of the endogenous actin 5 encoding gene, while the Actin5 promoter sequence serves as 5´ homologous flank. Prior to transformation the plasmid was linearized using *Hin*dIII.

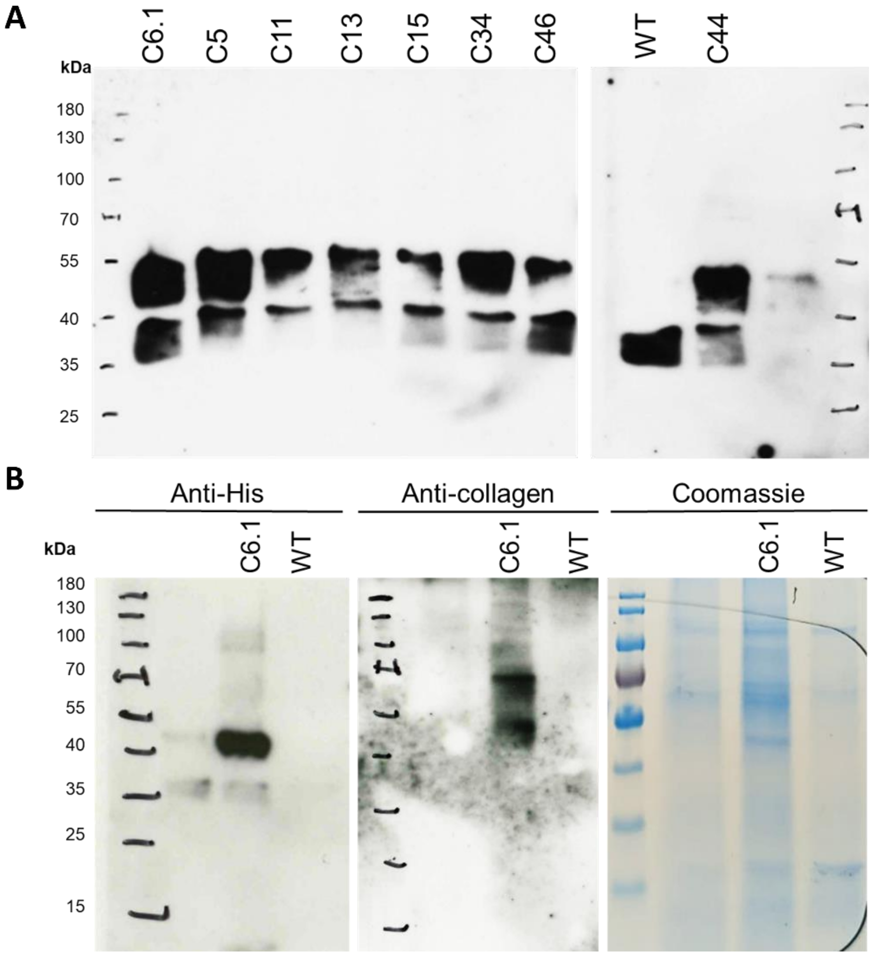

Supplementary Figure S3 Uncropped immunodetection images and Coomassie stained SDS-PAGE gel image corresponding to Figure 2
A Corresponds to **Figure 2A** and **B** corresponds to **Figure 2B**.

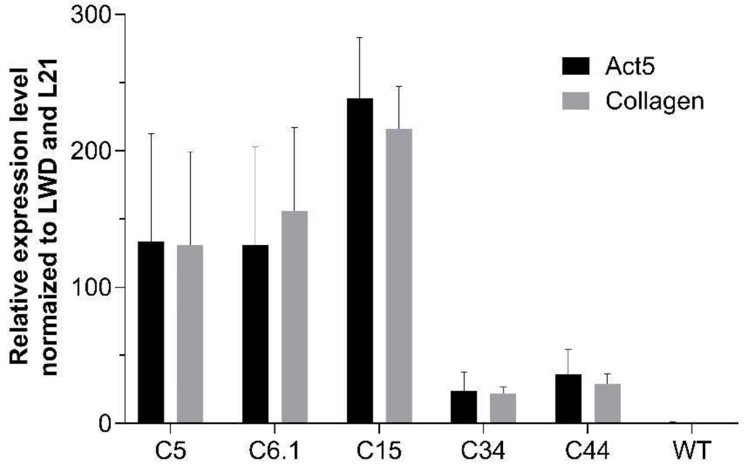

Supplementary Figure S4 Quantitative analysis of construct copy numbers in selected moss lines

The five selected collagen polypeptide-producing lines and an equally treated WT control were investigated for the number of integrated expression constructs by qPCR. Copy numbers were analysed using the primer pairs targeting the Actin5 promotor (Act5) and the collagen CDS (Collagen). Internal normalization to single integration signals was performed with signals obtained by two primer pairs targeting the endogenous single copy CLF gene and single integration signals for the Actin5 promoter were gained from the wild type (WT). Bars represent normalized mean values ± SD from n = 3 technical replicates.

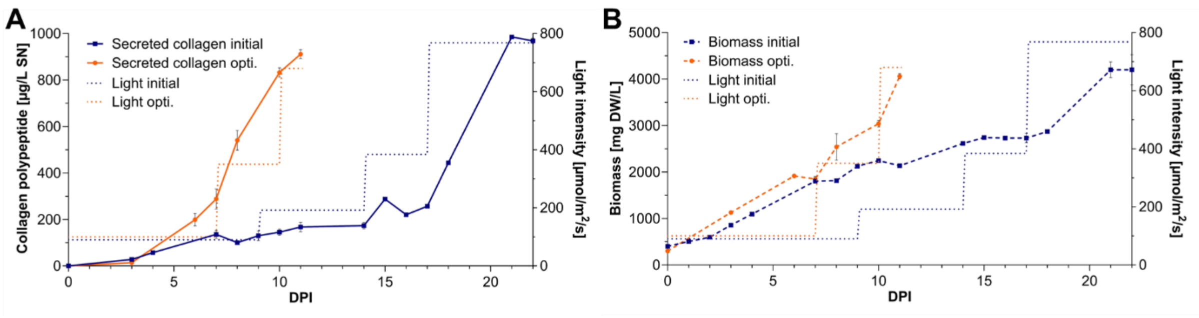

Supplementary Figure S5 Split version of Figure 5A, depicting parameters during 5 L photobioreactor runs of line C5 under initial and optimized conditions

A Secreted collagen polypeptide accumulation profiles (solid lines) and applied light intensity settings (dotted lines). Collagen concentrations were quantified by ELISA (mean ± SD, technical triplicates). **B** Moss biomass accumulation (dashed lines) and applied light intensity settings (dotted lines). Biomass quantification was performed by dry weight measurements (mean ± SD, technical duplicates).

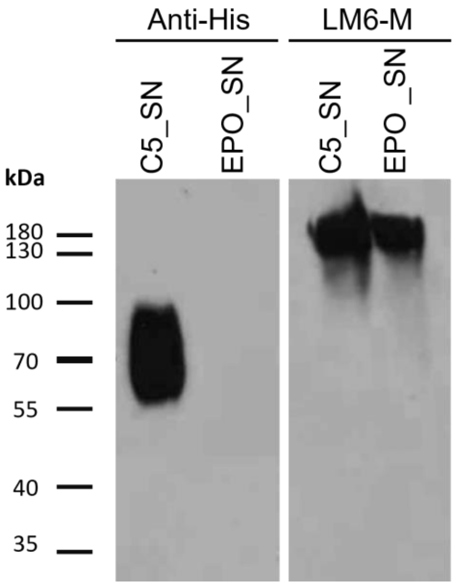

Supplementary Figure S6 Immunodetection of precipitated bioreactor culture supernatants

Precipitated and blotted bioreactor culture supernatants (SN) from the rhEPO-producing moss line *Δ*galt1 (EPO; Bohlender et al. 2022) and from line C5 were analysed with an anti-His antibody and the anti-1,5-α-L-arabinan antibody LM6-M. While LM6-M detection revealed the presence of arabinogalactan proteins in both samples at an apparent molecular weight of approximately 180 kDa (Lee et al. 2005; Bohlender et al. 2022), no corresponding signals were observed in the 55–90 kDa molecular weight range, where the secreted collagen peptide, as detected by the anti-His antibody, accumulates.

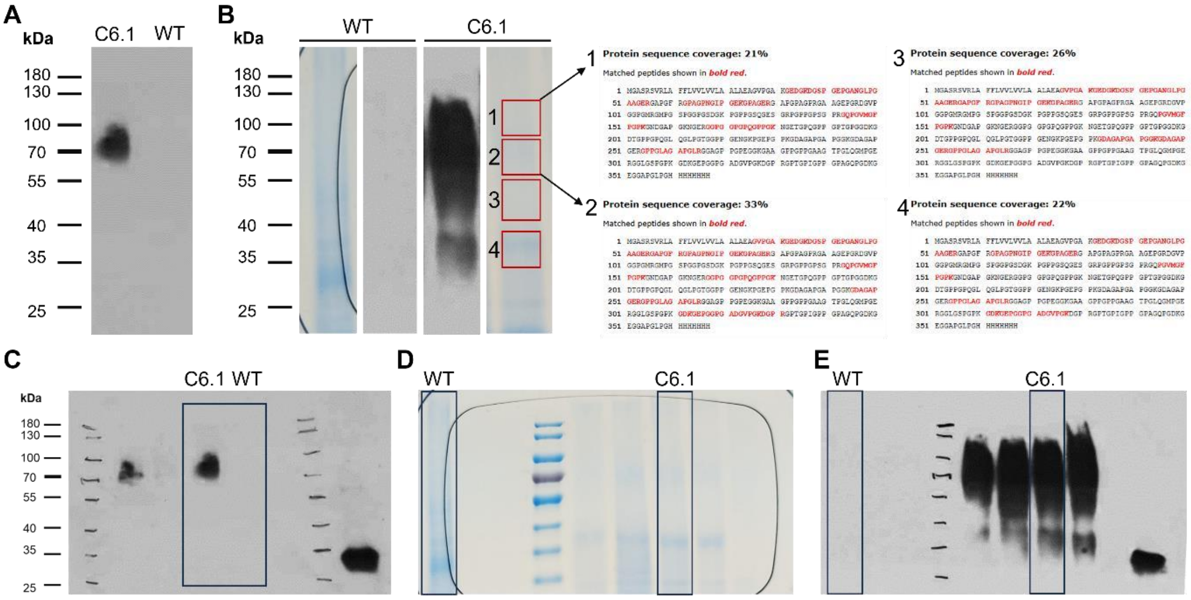

Supplementary Figure S7 Immunodetection from bioreactor culture supernatants, corresponding SDS-PAGE gels, mass spectrometrically determined sequence coverages of the recombinant collagen polypeptide, and uncropped images

A Anti-His antibody-based immunodetection of SDS-PAGE–separated and PVDF-transferred, non-precipitated bioreactor culture supernatants (40 µL per lane) from line C6.1 and the wild type (WT). **B** Coomassie-stained SDS-PAGE gels and corresponding anti-His immunodetection of 1.5 mL acetone-precipitated bioreactor culture supernatants from line C6.1 after 16 days of cultivation, compared to an equally treated WT control. Gel regions corresponding to immunodetection signals (marked as 1-4) were excised and analysed by mass spectrometry. The identified sequence coverages of the recombinant collagen polypeptide are marked in red. **C** Uncropped immunodetection image corresponding to **Supplementary Figure S7A**. **D** Uncropped SDS-PAGE gel image corresponding to **Supplementary Figure S7B**. **E** Uncropped immunodetection image corresponding to **Figure 6B** and **Supplementary Figure S7B**.

Supplementary Table S1 Underrepresented codons that were exchanged by overrepresented ones in the optimized CDS used for collagen production

These selections are based the codon usage tables described by Hiss et al. (2017) and Nakamura et al. (2000).

**Amino acid**	**Underrepresented codon**	**Overrepresented codon**
Cys	TGT	TGC
Glu	GAA	GAG
Phe	TTT	TTC
His	CAT	CAC
Lys	AAA	AAG
Asn	AAT	AAC
Gln	CAA	CAG
Tyr	TAT	TAC

Supplementary Table S2 Primers used for quantitative PCR

Primer	Sequence	Target	Experiment
Collagen_qP_F	AGAGAAGGGACCTGCTGGAG	Collagen CDS	qPCR, qRTPCR
Collagen_qP_R	GGAGATCCAGGCATTCCTCTC	Collagen CDS	qPCR, qRTPCR
qPCR LWD fwd	CACTGCAGGAGCTGAAATCAATC	LWD CDS	qRT-PCR
qPCR LWD rev	CGTAGTATCTGGAGCTTGGTGGA	LWD CDS	qRT-PCR
c45-tg2F	ACGCACCGGCATCGT	L21 CDS	qRT-PCR
c45-tg2R	TGCTTGTTCATCACGACACCA	L21 CDS	qRT-PCR
pPCLF_5915_qf	AGCAATGTCCGTGCCTACTT	CLF CDS	qPCR
pPCLF_5981_qr	TTGTAAGAATCACTCACCCACAG	CLF CDS	qPCR
pPCLF_7739_qf	GTATTGGCGATCCCACTCTT	CLF CDS	qPCR
pPCLF_7804_qr	GCATAAAATAGGTCACAGATTGAGG	CLF CDS	qPCR
qhpt_f	ATACGAGGTCGCCAACATCT	hpt CDS	qPCR
qhpt_r	TGCCTCCGCTCGAAGTAG	hpt CDS	qPCR
Act5P_qPCR_fwd	GCTTGGTCCGAGGCTATTATT	Actin5 promoter	qPCR
Act5p_qPCR_rev	TAGGTGCAACCCGCTGTT	Actin5 promoter	qPCR

Supplementary Table S3 Quantitation summary of identified hydroxylated prolines in secreted collagen samples

Shown are the proline residues identified across the seven analysed measurements (raw files), including their respective G-X-Y motif and their corresponding amino acid positions in the recombinant collagen polypeptide (Position_Motif). Log_2-transformed peptide intensities were used as quantitative values. These were first normalized to the median intensity within each measurement and subsequently centred to the global median intensity across all measurements. For each identified hydroxyproline or its unmodified counterpart the quantitative values from each corresponding peptide isoform were summed within each measurement (Raw File). The relative proportion of modified versus unmodified prolines per peptide is given as a percentage.

Raw File	Position Motif	Type	Summed Intensity	Total Intensity	Percent
ELITE-RSLC037807	40_GSP	Mod.	20.8633	84.8853	24.58
ELITE-RSLC037807	40_GSP	Unmod.	64.0221	84.8853	75.42
ELITE-RSLC037807	43_GEP	Unmod.	84.8853	84.8853	100.00
ELITE-RSLC037807	49_GLP	Unmod.	84.8853	84.8853	100.00
ELITE-RSLC037807	63_GPA	Mod.	16.1702	16.1702	100.00
ELITE-RSLC037807	66_GPN	Mod.	16.1702	16.1702	100.00
ELITE-RSLC037807	70_GIP	Unmod.	34.5415	34.5415	100.00
ELITE-RSLC037807	75_GPA	Unmod.	35.5944	35.5944	100.00
ELITE-RSLC037807	94_GEP	Unmod.	18.7694	18.7694	100.00
ELITE-RSLC037807	100_GVP	Mod.	18.7694	53.3520	35.18
ELITE-RSLC037807	100_GVP	Unmod.	34.5826	53.3520	64.82
ELITE-RSLC037807	103_GGP	Mod.	18.7694	36.9129	50.85
ELITE-RSLC037807	103_GGP	Unmod.	18.1435	36.9129	49.15
ELITE-RSLC037807	145_GQP	Unmod.	23.2473	23.2473	100.00
ELITE-RSLC037807	153_GPK	Unmod.	23.2473	23.2473	100.00
ELITE-RSLC037807	250_GAP	Mod.	17.6325	17.6325	100.00
ELITE-RSLC037807	255_GPP	Unmod.	20.5607	20.5607	100.00
ELITE-RSLC037807	256_GPP	Unmod.	61.5526	61.5526	100.00
ELITE-RSLC037807	262_GAP	Mod.	38.1931	38.1931	100.00
ELITE-RSLC037807	316_GEP	Unmod.	33.5776	33.5776	100.00
ELITE-RSLC037807	319_GGP	Unmod.	33.5776	33.5776	100.00
ELITE-RSLC037807	325_GVP	Unmod.	33.5776	33.5776	100.00
ELITE-RSLC037809	28_GVP	Mod.	66.4161	139.2935	47.68
ELITE-RSLC037809	28_GVP	Unmod.	72.8774	139.2935	52.32
ELITE-RSLC037809	40_GSP	Mod.	133.3001	529.5811	25.17
ELITE-RSLC037809	40_GSP	Unmod.	396.2810	529.5811	74.83

ELITE-RSLC037809	43_GEP	Unmod.	599.2329	599.2329	100.00
ELITE-RSLC037809	49_GLP	Mod.	58.1106	599.2329	9.70
ELITE-RSLC037809	49_GLP	Unmod.	541.1223	599.2329	90.30
ELITE-RSLC037809	58_GAP	Unmod.	20.2887	20.2887	100.00
ELITE-RSLC037809	63_GPA	Mod.	39.3541	58.2412	67.57
ELITE-RSLC037809	63_GPA	Unmod.	18.8871	58.2412	32.43

ELITE-RSLC037809	66_GPN	Mod.	39.3541	98.2693	40.05
ELITE-RSLC037809	66_GPN	Unmod.	58.9152	98.2693	59.95
ELITE-RSLC037809	70_GIP	Unmod.	176.4821	176.4821	100.00
ELITE-RSLC037809	75_GPA	Unmod.	176.2793	176.2793	100.00
ELITE-RSLC037809	94_GEP	Mod.	23.9800	66.7997	35.90
ELITE-RSLC037809	94_GEP	Unmod.	42.8198	66.7997	64.10
ELITE-RSLC037809	100_GVP	Mod.	23.9800	61.5583	38.95
ELITE-RSLC037809	100_GVP	Unmod.	37.5784	61.5583	61.05
ELITE-RSLC037809	103_GGP	Mod.	23.9800	77.9768	30.75
ELITE-RSLC037809	103_GGP	Unmod.	53.9969	77.9768	69.25
ELITE-RSLC037809	145_GQP	Mod.	44.9735	93.5253	48.09
ELITE-RSLC037809	145_GQP	Unmod.	48.5518	93.5253	51.91
ELITE-RSLC037809	151_GFP	Mod.	21.0235	88.6469	23.72
ELITE-RSLC037809	151_GFP	Unmod.	67.6234	88.6469	76.28
ELITE-RSLC037809	153_GPK	Mod.	21.0235	161.6960	13.00
ELITE-RSLC037809	153_GPK	Unmod.	140.6725	161.6960	87.00
ELITE-RSLC037809	169_GPG	Unmod.	17.9621	17.9621	100.00
ELITE-RSLC037809	172_GPG	Unmod.	17.9621	17.9621	100.00
ELITE-RSLC037809	174_GPQ	Unmod.	17.9621	17.9621	100.00
ELITE-RSLC037809	177_GPP	Unmod.	17.9621	17.9621	100.00
ELITE-RSLC037809	178_GPP	Unmod.	17.9621	17.9621	100.00
ELITE-RSLC037809	241_GAP	Unmod.	16.0661	16.0661	100.00
ELITE-RSLC037809	250_GAP	Mod.	68.8330	84.9600	81.02
ELITE-RSLC037809	250_GAP	Unmod.	16.1270	84.9600	18.98
ELITE-RSLC037809	255_GPP	Unmod.	90.1647	90.1647	100.00
ELITE-RSLC037809	256_GPP	Mod.	26.2192	160.7007	16.32
ELITE-RSLC037809	256_GPP	Unmod.	134.4815	160.7007	83.68
ELITE-RSLC037809	262_GAP	Mod.	93.2291	113.0045	82.50
ELITE-RSLC037809	262_GAP	Unmod.	19.7754	113.0045	17.50
ELITE-RSLC037809	283_GPP	Unmod.	20.1412	20.1412	100.00
ELITE-RSLC037809	285_GPP	Unmod.	20.1412	20.1412	100.00
ELITE-RSLC037809	286_GPP	Unmod.	20.1412	20.1412	100.00
ELITE-RSLC037809	292_GTP	Unmod.	20.1412	20.1412	100.00
ELITE-RSLC037809	298_GMP	Unmod.	20.1412	20.1412	100.00
ELITE-RSLC037809	307_GSP	Unmod.	19.6213	19.6213	100.00
ELITE-RSLC037809	309_GPK	Unmod.	19.6213	19.6213	100.00
ELITE-RSLC037809	316_GEP	Unmod.	289.2604	289.2604	100.00

ELITE-RSLC037809	319_GGP	Mod.	21.3337	290.5687	7.34
ELITE-RSLC037809	319_GGP	Unmod.	269.2350	290.5687	92.66
ELITE-RSLC037809	325_GVP	Mod.	57.9244	309.5610	18.71
ELITE-RSLC037809	325_GVP	Unmod.	251.6366	309.5610	81.29
ELITE-RSLC037809	330_GPR	Unmod.	193.2421	193.2421	100.00
ELITE-RSLC037842	28_GVP	Mod.	16.5849	101.8921	16.28
ELITE-RSLC037842	28_GVP	Unmod.	85.3072	101.8921	83.72
ELITE-RSLC037842	40_GSP	Mod.	39.8071	204.3013	19.48
ELITE-RSLC037842	40_GSP	Unmod.	164.4942	204.3013	80.52
ELITE-RSLC037842	43_GEP	Mod.	19.7206	266.1995	7.41
ELITE-RSLC037842	43_GEP	Unmod.	246.4790	266.1995	92.59
ELITE-RSLC037842	49_GLP	Unmod.	284.1138	284.1138	100.00
ELITE-RSLC037842	63_GPA	Mod.	35.2337	35.2337	100.00
ELITE-RSLC037842	66_GPN	Mod.	35.2337	52.2873	67.38
ELITE-RSLC037842	66_GPN	Unmod.	17.0536	52.2873	32.62
ELITE-RSLC037842	70_GIP	Unmod.	103.9241	103.9241	100.00
ELITE-RSLC037842	75_GPA	Mod.	17.7395	87.3611	20.31
ELITE-RSLC037842	75_GPA	Unmod.	69.6216	87.3611	79.69
ELITE-RSLC037842	103_GGP	Unmod.	19.7747	19.7747	100.00
ELITE-RSLC037842	145_GQP	Unmod.	17.4944	17.4944	100.00
ELITE-RSLC037842	151_GFP	Mod.	17.4944	81.1963	21.55
ELITE-RSLC037842	151_GFP	Unmod.	63.7019	81.1963	78.45
ELITE-RSLC037842	153_GPK	Mod.	17.4944	100.1879	17.46
ELITE-RSLC037842	153_GPK	Unmod.	82.6935	100.1879	82.54
ELITE-RSLC037842	169_GPG	Unmod.	31.8294	31.8294	100.00
ELITE-RSLC037842	172_GPG	Unmod.	31.8294	31.8294	100.00
ELITE-RSLC037842	174_GPQ	Unmod.	31.8294	31.8294	100.00
ELITE-RSLC037842	177_GPP	Unmod.	31.8294	31.8294	100.00
ELITE-RSLC037842	178_GPP	Unmod.	31.8294	31.8294	100.00
ELITE-RSLC037842	250_GAP	Mod.	21.4815	21.4815	100.00
ELITE-RSLC037842	255_GPP	Unmod.	66.0051	66.0051	100.00
ELITE-RSLC037842	256_GPP	Mod.	24.9956	87.4866	28.57
ELITE-RSLC037842	256_GPP	Unmod.	62.4910	87.4866	71.43
ELITE-RSLC037842	262_GAP	Mod.	68.8090	87.4866	78.65
ELITE-RSLC037842	262_GAP	Unmod.	18.6776	87.4866	21.35
ELITE-RSLC037842	282_GPP	Unmod.	17.7921	17.7921	100.00
ELITE-RSLC037842	283_GPP	Unmod.	17.7921	17.7921	100.00

ELITE-RSLC037842	285_GPP	Unmod.	17.7921	17.7921	100.00
ELITE-RSLC037842	286_GPP	Unmod.	17.7921	17.7921	100.00
ELITE-RSLC037842	292_GTP	Unmod.	17.7921	17.7921	100.00
ELITE-RSLC037842	298_GMP	Unmod.	17.7921	17.7921	100.00
ELITE-RSLC037842	316_GEP	Unmod.	81.4029	81.4029	100.00
ELITE-RSLC037842	319_GGP	Unmod.	81.4029	81.4029	100.00
ELITE-RSLC037842	325_GVP	Unmod.	66.5101	66.5101	100.00
ELITE-RSLC037842	330_GPR	Unmod.	31.9191	31.9191	100.00
ELITE-RSLC037844	40_GSP	Unmod.	105.4740	105.4740	100.00
ELITE-RSLC037844	43_GEP	Unmod.	105.4740	105.4740	100.00
ELITE-RSLC037844	49_GLP	Unmod.	85.9872	85.9872	100.00
ELITE-RSLC037844	63_GPA	Mod.	18.1163	18.1163	100.00
ELITE-RSLC037844	66_GPN	Unmod.	34.2999	34.2999	100.00
ELITE-RSLC037844	70_GIP	Mod.	18.1163	51.7510	35.01
ELITE-RSLC037844	70_GIP	Unmod.	33.6348	51.7510	64.99
ELITE-RSLC037844	75_GPA	Unmod.	35.1084	35.1084	100.00
ELITE-RSLC037844	94_GEP	Unmod.	37.5798	37.5798	100.00
ELITE-RSLC037844	100_GVP	Mod.	19.5219	19.5219	100.00
ELITE-RSLC037844	103_GGP	Mod.	19.5219	19.5219	100.00
ELITE-RSLC037844	145_GQP	Mod.	19.4177	44.3263	43.81
ELITE-RSLC037844	145_GQP	Unmod.	24.9086	44.3263	56.19
ELITE-RSLC037844	151_GFP	Unmod.	40.6166	40.6166	100.00
ELITE-RSLC037844	153_GPK	Mod.	19.4177	62.7464	30.95
ELITE-RSLC037844	153_GPK	Unmod.	43.3287	62.7464	69.05
ELITE-RSLC037844	169_GPG	Unmod.	16.1934	16.1934	100.00
ELITE-RSLC037844	172_GPG	Unmod.	16.1934	16.1934	100.00
ELITE-RSLC037844	174_GPQ	Unmod.	16.1934	16.1934	100.00
ELITE-RSLC037844	177_GPP	Unmod.	16.1934	16.1934	100.00
ELITE-RSLC037844	178_GPP	Unmod.	16.1934	16.1934	100.00
ELITE-RSLC037844	250_GAP	Mod.	43.4220	43.4220	100.00
ELITE-RSLC037844	255_GPP	Unmod.	40.4483	40.4483	100.00
ELITE-RSLC037844	256_GPP	Unmod.	86.8307	86.8307	100.00
ELITE-RSLC037844	262_GAP	Mod.	43.1026	83.8703	51.39
ELITE-RSLC037844	262_GAP	Unmod.	40.7677	83.8703	48.61
ELITE-RSLC037844	316_GEP	Unmod.	33.4864	33.4864	100.00
ELITE-RSLC037844	319_GGP	Unmod.	33.4864	33.4864	100.00
ELITE-RSLC037844	325_GVP	Unmod.	33.4864	33.4864	100.00

ELITE-RSLC037846	40_GSP	Mod.	19.0729	126.0361	15.13
ELITE-RSLC037846	40_GSP	Unmod.	106.9632	126.0361	84.87
ELITE-RSLC037846	43_GEP	Mod.	40.7095	177.4290	22.94
ELITE-RSLC037846	43_GEP	Unmod.	136.7195	177.4290	77.06
ELITE-RSLC037846	49_GLP	Unmod.	177.4290	177.4290	100.00
ELITE-RSLC037846	63_GPA	Mod.	57.5090	130.3027	44.13
ELITE-RSLC037846	63_GPA	Unmod.	72.7938	130.3027	55.87
ELITE-RSLC037846	66_GPN	Mod.	54.8535	164.7228	33.30
ELITE-RSLC037846	66_GPN	Unmod.	109.8693	164.7228	66.70
ELITE-RSLC037846	70_GIP	Mod.	41.4313	115.0897	36.00
ELITE-RSLC037846	70_GIP	Unmod.	73.6584	115.0897	64.00
ELITE-RSLC037846	75_GPA	Mod.	41.4313	110.7541	37.41
ELITE-RSLC037846	75_GPA	Unmod.	69.3228	110.7541	62.59
ELITE-RSLC037846	94_GEP	Unmod.	18.3197	18.3197	100.00
ELITE-RSLC037846	103_GGP	Unmod.	18.3197	18.3197	100.00
ELITE-RSLC037846	145_GQP	Mod.	43.6728	94.3200	46.30
ELITE-RSLC037846	145_GQP	Unmod.	50.6472	94.3200	53.70
ELITE-RSLC037846	151_GFP	Mod.	20.9698	20.9698	100.00
ELITE-RSLC037846	153_GPK	Mod.	20.9698	115.2898	18.19
ELITE-RSLC037846	153_GPK	Unmod.	94.3200	115.2898	81.81
ELITE-RSLC037846	169_GPG	Unmod.	17.8969	17.8969	100.00
ELITE-RSLC037846	172_GPG	Unmod.	17.8969	17.8969	100.00
ELITE-RSLC037846	174_GPQ	Unmod.	17.8969	17.8969	100.00
ELITE-RSLC037846	177_GPP	Unmod.	17.8969	17.8969	100.00
ELITE-RSLC037846	178_GPP	Unmod.	17.8969	17.8969	100.00
ELITE-RSLC037846	238_GAP	Unmod.	15.7655	15.7655	100.00
ELITE-RSLC037846	241_GAP	Mod.	15.7655	31.5955	49.90
ELITE-RSLC037846	241_GAP	Unmod.	15.8301	31.5955	50.10
ELITE-RSLC037846	250_GAP	Mod.	15.7655	38.0657	41.42
ELITE-RSLC037846	250_GAP	Unmod.	22.3003	38.0657	58.58
ELITE-RSLC037846	255_GPP	Mod.	22.3003	45.4473	49.07
ELITE-RSLC037846	255_GPP	Unmod.	23.1470	45.4473	50.93
ELITE-RSLC037846	256_GPP	Mod.	22.3003	87.7776	25.41
ELITE-RSLC037846	256_GPP	Unmod.	65.4773	87.7776	74.59
ELITE-RSLC037846	262_GAP	Mod.	45.4473	45.4473	100.00
ELITE-RSLC037846	282_GPP	Unmod.	18.6874	18.6874	100.00
ELITE-RSLC037846	283_GPP	Unmod.	18.6874	18.6874	100.00

ELITE-RSLC037846	285_GPP	Unmod.	18.6874	18.6874	100.00
ELITE-RSLC037846	286_GPP	Unmod.	18.6874	18.6874	100.00
ELITE-RSLC037846	298_GMP	Unmod.	18.6874	18.6874	100.00
ELITE-RSLC037846	316_GEP	Unmod.	124.4596	124.4596	100.00
ELITE-RSLC037846	319_GGP	Unmod.	124.4596	124.4596	100.00
ELITE-RSLC037846	325_GVP	Mod.	32.7884	124.4596	26.34
ELITE-RSLC037846	325_GVP	Unmod.	91.6712	124.4596	73.66
ELITE-RSLC037848	28_GVP	Unmod.	41.7938	41.7938	100.00
ELITE-RSLC037848	40_GSP	Unmod.	228.8501	228.8501	100.00
ELITE-RSLC037848	43_GEP	Mod.	18.8140	250.1121	7.52
ELITE-RSLC037848	43_GEP	Unmod.	231.2981	250.1121	92.48
ELITE-RSLC037848	49_GLP	Unmod.	268.6751	268.6751	100.00
ELITE-RSLC037848	58_GAP	Unmod.	17.1999	17.1999	100.00
ELITE-RSLC037848	63_GPA	Mod.	18.1023	37.4342	48.36
ELITE-RSLC037848	63_GPA	Unmod.	19.3319	37.4342	51.64
ELITE-RSLC037848	66_GPN	Mod.	37.4342	91.2268	41.03
ELITE-RSLC037848	66_GPN	Unmod.	53.7926	91.2268	58.97
ELITE-RSLC037848	70_GIP	Mod.	19.3319	126.5739	15.27
ELITE-RSLC037848	70_GIP	Unmod.	107.2420	126.5739	84.73
ELITE-RSLC037848	75_GPA	Mod.	19.3319	129.3813	14.94
ELITE-RSLC037848	75_GPA	Unmod.	110.0494	129.3813	85.06
ELITE-RSLC037848	103_GGP	Unmod.	21.3252	21.3252	100.00
ELITE-RSLC037848	145_GQP	Mod.	40.3388	108.1789	37.29
ELITE-RSLC037848	145_GQP	Unmod.	67.8401	108.1789	62.71
ELITE-RSLC037848	151_GFP	Unmod.	57.5944	57.5944	100.00
ELITE-RSLC037848	153_GPK	Unmod.	119.7812	119.7812	100.00
ELITE-RSLC037848	169_GPG	Unmod.	31.5013	31.5013	100.00
ELITE-RSLC037848	172_GPG	Unmod.	31.5013	31.5013	100.00
ELITE-RSLC037848	174_GPQ	Unmod.	31.5013	31.5013	100.00
ELITE-RSLC037848	177_GPP	Unmod.	31.5013	31.5013	100.00
ELITE-RSLC037848	178_GPP	Unmod.	31.5013	31.5013	100.00
ELITE-RSLC037848	250_GAP	Mod.	20.6807	20.6807	100.00
ELITE-RSLC037848	255_GPP	Unmod.	64.5775	64.5775	100.00
ELITE-RSLC037848	256_GPP	Mod.	20.6807	115.6182	17.89
ELITE-RSLC037848	256_GPP	Unmod.	94.9376	115.6182	82.11
ELITE-RSLC037848	262_GAP	Mod.	68.8397	68.8397	100.00
ELITE-RSLC037848	282_GPP	Unmod.	20.0323	20.0323	100.00

ELITE-RSLC037848	283_GPP	Unmod.	20.0323	20.0323	100.00
ELITE-RSLC037848	285_GPP	Unmod.	20.0323	20.0323	100.00
ELITE-RSLC037848	286_GPP	Unmod.	20.0323	20.0323	100.00
ELITE-RSLC037848	292_GTP	Unmod.	20.0323	20.0323	100.00
ELITE-RSLC037848	298_GMP	Unmod.	20.0323	20.0323	100.00
ELITE-RSLC037848	307_GSP	Mod.	35.7435	35.7435	100.00
ELITE-RSLC037848	309_GPK	Mod.	35.7435	35.7435	100.00
ELITE-RSLC037848	316_GEP	Unmod.	106.9230	106.9230	100.00
ELITE-RSLC037848	319_GGP	Mod.	17.9221	177.4607	10.10
ELITE-RSLC037848	319_GGP	Unmod.	159.5386	177.4607	89.90
ELITE-RSLC037848	325_GVP	Unmod.	177.4607	177.4607	100.00
ELITE-RSLC037848	330_GPR	Unmod.	56.7676	56.7676	100.00
ORBI-RSLC037282	40_GSP	Unmod.	50.9065	50.9065	100.00
ORBI-RSLC037282	43_GEP	Unmod.	50.9065	50.9065	100.00
ORBI-RSLC037282	49_GLP	Unmod.	50.9065	50.9065	100.00
ORBI-RSLC037282	151_GFP	Unmod.	21.3525	21.3525	100.00
ORBI-RSLC037282	153_GPK	Unmod.	21.3525	21.3525	100.00
ORBI-RSLC037282	169_GPG	Unmod.	16.0640	16.0640	100.00
ORBI-RSLC037282	172_GPG	Unmod.	16.0640	16.0640	100.00
ORBI-RSLC037282	174_GPQ	Unmod.	16.0640	16.0640	100.00
ORBI-RSLC037282	177_GPP	Unmod.	16.0640	16.0640	100.00
ORBI-RSLC037282	178_GPP	Unmod.	16.0640	16.0640	100.00
ORBI-RSLC037282	255_GPP	Unmod.	22.7614	22.7614	100.00
ORBI-RSLC037282	256_GPP	Unmod.	22.7614	22.7614	100.00
ORBI-RSLC037282	262_GAP	Unmod.	22.7614	22.7614	100.00
ORBI-RSLC037282	270_GPP	Unmod.	14.6829	14.6829	100.00
ORBI-RSLC037282	271_GPP	Unmod.	14.6829	14.6829	100.00
ORBI-RSLC037282	273_GPE	Unmod.	14.6829	14.6829	100.00
ORBI-RSLC037282	282_GPP	Unmod.	41.6391	41.6391	100.00
ORBI-RSLC037282	283_GPP	Unmod.	41.6391	41.6391	100.00
ORBI-RSLC037282	285_GPP	Unmod.	60.3819	60.3819	100.00
ORBI-RSLC037282	286_GPP	Unmod.	60.3819	60.3819	100.00
ORBI-RSLC037282	292_GTP	Unmod.	41.6391	41.6391	100.00
ORBI-RSLC037282	298_GMP	Unmod.	60.3819	60.3819	100.00

136
